# Vitamin D Deficiency and the Risk of Cerebrovascular Disease

**DOI:** 10.3390/antiox9040327

**Published:** 2020-04-17

**Authors:** Hyun Ah Kim, Andrea Perrelli, Alberto Ragni, Francesca Retta, T. Michael De Silva, Christopher G. Sobey, Saverio Francesco Retta

**Affiliations:** 1 Department of Physiology, Anatomy & Microbiology and Centre for Cardiovascular Biology and Disease Research, School of Life Sciences, La Trobe University, Bundoora 3086, Australia; H.Kim2@latrobe.edu.au (H.A.K.); T.DeSilva@latrobe.edu.au (T.M.D.S.); 2 Cardiovascular Disease Program, Biomedicine Discovery Institute and Department of Pharmacology, Monash University, Clayton 3800, Australia; 3 Department of Clinical and Biological Sciences, University of Torino, Orbassano, 10043 Torino, Italy; andrea.perrelli@unito.it; 4 CCM Italia Research Network, National Coordination Center at the Department of Clinical and Biological Sciences, University of Torino, Orbassano, 10043 Torino, Italy; 5 Oncological Endocrinology Unit, Department of Medical Sciences, Città della Salute e della Scienza Hospital, University of Torino, 10126 Torino, Italy; alberto.ragni@edu.unito.it (A.R.); francesca.retta@edu.unito.it (F.R.)

**Keywords:** cerebrovascular disease, stroke, cerebral cavernous malformation (CCM), vitamin D, oxidative stress, inflammation, endothelial dysfunction, redox homeostasis and signaling, autophagy, antioxidant and anti-inflammatory defenses

## Abstract

Vitamin D deficiency has been clearly linked to major chronic diseases associated with oxidative stress, inflammation, and aging, including cardiovascular and neurodegenerative diseases, diabetes, and cancer. In particular, the cardiovascular system appears to be highly sensitive to vitamin D deficiency, as this may result in endothelial dysfunction and vascular defects via multiple mechanisms. Accordingly, recent research developments have led to the proposal that pharmacological interventions targeting either vitamin D deficiency or its key downstream effects, including defective autophagy and abnormal pro-oxidant and pro-inflammatory responses, may be able to limit the onset and severity of major cerebrovascular diseases, such as stroke and cerebrovascular malformations. Here we review the available evidence supporting the role of vitamin D in preventing or limiting the development of these cerebrovascular diseases, which are leading causes of disability and death all over the world.

## 1. Sources, Metabolism, and Pleiotropic Functions of Vitamin D

The term vitamin D refers to a group of lipid-soluble secosteroid compounds with pro-hormone activities, of which five forms have been described: vitamin D_1_, D_2_, D_3_, D_4_, and D_5_. Among these, the most important for human biology are vitamin D_2_ (also known as ergocalciferol), which is produced in plants and fungi from the precursor ergosterol upon exposure to the sun’s ultraviolet B (UVB) rays, and vitamin D_3_ (also known as cholecalciferol), which is mainly produced in the skin from the precursor 7-dehydrocholesterol (7-DHC) upon exposure to UVB rays and may also be obtained from animal sources or dietary supplements. Both vitamins D_2_ and D_3_ are transported in the blood by carrier proteins, mainly by vitamin D binding protein (VDBP), but also by albumin and lipoproteins, and distributed to other tissues (primarily the liver). In the liver, they are hydroxylated at C-25 by 25-hydroxylase enzymes of the cytochrome P450 monooxygenase (CYP) family (mostly but not exclusively CYP2R1 and CYP27A1) to generate the main circulating form of vitamin D: 25-hydroxy-vitamin D (25(OH)D). The 25(OH)D is then transported by vitamin D binding proteins via the blood to the kidneys, where it is internalized by renal proximal tubular cells through receptor (megalin)-mediated endocytosis. There it undergoes a further hydroxylation at C-1 by the mitochondrial 1-alpha-hydroxylase enzyme (CYP27B1), to produce the hormonally active form of vitamin D, 1,25-dihydroxy-vitamin D (1,25(OH)_2_D), which is responsible for most, if not all of its biological actions [1,2,3,4]. Two forms of 1,25(OH)_2_D exist: 1,25(OH)_2_D_3_ (calcitriol) and 1,25(OH)_2_D_2_ (ercalcitriol), which are derived from cholecalciferol and ergocalciferol, respectively. Although the kidneys are the major source of circulating 1,25(OH)_2_D, a number of other tissues also express the CYP27B1 enzyme, which uniquely possesses 25(OH)D 1-alpha-hydroxylase activity. Inactivation and catabolism of both 25(OH)D and 1,25(OH)_2_D are specifically mediated by the 24-hydroxylase activity of the mitochondrial CYP24A1 enzyme [2].

**Figure 1:**
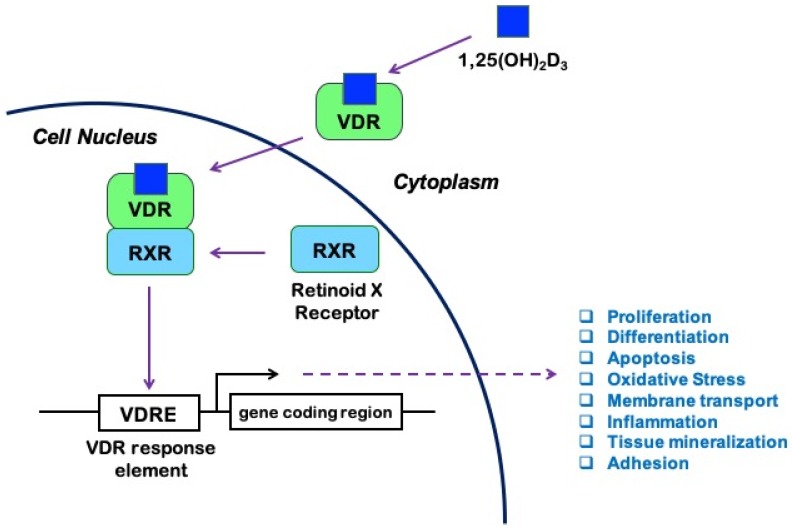
Vitamin D signaling pathway: 1,25-hydroxyvitamin D (1,25(OH)_2_D_3_), also known as calcitriol, binds to the vitamin D receptor (VDR) and promotes its heterodimerization with the retinoid X receptor (RXR). The activated VDR/RXR heterodimer then recruits coregulator complexes and binds to the vitamin D response elements (VDRE) in the promoters of a large number of genes involved in fundamental processes, including cell survival and immune response to injury, thus modulating their transcription and subsequent effects in a ligand-dependent manner.

It is known that 1,25(OH)_2_D exerts its biological effects by binding to and activating the vitamin D receptor (VDR), a member of the ligand-regulated nuclear receptor superfamily of transcription factors widely distributed in the body, expressed by leukocytes [5], endothelial cells [6], astrocytes, and neurons [7]. Both forms of 1,25(OH)_2_D can activate the VDR, with similar affinity [2]. Upon activation by ligand binding, VDR heterodimerizes with the retinoid X receptor (RXR) to form a transcriptionally active complex [1,8,9]. Formation of the VDR/RXR-heterodimer and its binding to DNA is essential for the regulation of gene transcription by 1,25(OH)_2_D [9]. In particular, the VDR/RXR complex binds vitamin D response elements (VDREs), which are specific promoter sequences. Co-regulator factors are then recruited to either increase or suppress the transcription of various target genes, including genes involved in cell proliferation, differentiation, apoptosis, inflammation, and oxidative stress [10] (Figure 1).

VDR is expressed in more than 30 target tissues in humans [11], and a genome-wide analysis revealed more than 1000 VDR-specific genomic binding sites in most tissues, suggesting that the transcriptionally active form of vitamin D influences the expression of many genes likely to be relevant for human health and disease [12]. Furthermore, lessons from VDR and CYP27B1 null mice indicate that VDR may act either dependently or independently of 1,25(OH)_2_D. Thus, multiple receptors and ligands may participate in the vitamin D endocrine system [1,3,13], in addition to non-genomic actions via unclear mechanisms [14,15,16]. Indeed, consistent with the multiple biological functions of the active form of vitamin D, there is evidence that VDR, which is normally localized in the nucleus and associated with gene transcription, may also be present in the plasma membrane and mediate rapid responses to 1,25(OH)_2_D [11,17].

Vitamin D plays a pivotal role in bone metabolism via calcium and phosphate homeostasis, whereby it stimulates calcium absorption and reabsorption in the intestine and the kidneys, respectively; it also contributes to the formation and resorption of bone tissue by promoting the differentiation of osteoblasts and regulating the effects by other bone-active molecules [16,18].

The ubiquitous expression of the VDR and the CYP27B1 enzyme has led to speculation that vitamin D may exert physiological roles other than via calcium-phosphate homeostasis (i.e., non-classical roles) [14,19]. Indeed, the physiological importance of vitamin D extends far beyond the regulation of calcium homeostasis and bone metabolism. Vitamin D can regulate secretion of some hormones, including parathyroid hormone (PTH), insulin, and fibroblast growth factor 23 (FGF23), and the effects on PTH secretion are facilitated by using vitamin D analogues in the clinical treatment of secondary hyperparathyroidism [20]. Vitamin D also plays an important role in regulating both innate and adaptive immunity. For example, activated monocytes express CYP27B1 to produce 1,25(OH)_2_D and induce antimicrobial peptides, and vitamin D suppresses the proliferation of both B and T lymphocytes, particularly the T helper-1 and-17 cells that are capable of activating macrophages [14]. On the other hand, regulatory T lymphocytes are increased by 1,25(OH)_2_D [21]. Consistent with the suppressive effect of vitamin D on the adaptive immune system, vitamin D deficiency and VDR polymorphisms are associated with increased risk of both systemic and organ-specific autoimmune diseases [22]. Moreover, an intervention trial has demonstrated that vitamin D could reduce the incidence of type 1 diabetes mellitus in Finnish infants (a population at high risk of developing autoimmune diseases) [23]. Vitamin D has also been shown to exert anti-proliferative and pro-differentiating effects on many cell types [24], leading to the evaluation of its potential anticancer activities in human trials [14,25].

The relationship between vitamin D and cardiovascular health has been extensively investigated. The cardiovascular system appears to be a target for vitamin D, with VDR and 1-alpha hydroxylase expressed in endothelial and vascular smooth muscle cells and cardiomyocytes, as well as in macrophages and T-cells, which is also highly relevant to VDR action in the cardiovascular system [26]. Vitamin D regulates the contractility of vascular smooth muscle cells via modulation of calcium influx, and appears to regulate endothelial function through its antioxidant effects and modulation of cell survival and autophagy [27,28]. Calcitriol has also been reported to be a negative regulator of the renin–angiotensin–aldosterone system [29], and thus vitamin D deficiency could contribute to endothelial dysfunction and the onset of cardiovascular diseases (CVDs). Various studies have indeed found an association between low circulating levels of 25(OH)D and the onset of hypertension, diabetes mellitus, and other CVDs [10,30,31,32,33]. Vitamin D could perhaps prevent endothelial dysfunction and atherosclerosis through its antioxidant and anti-inflammatory actions (e.g., inhibition of superoxide anion generation, modulation of cytokine secretion, and inhibition of monocyte adhesion and migration) [27,34,35,36,37]. More generally, as vitamin D deficiency is associated with many pathologies resembling those induced by defective autophagy, it has been suggested that autophagy plays a major role in the multiple health-promoting effects of vitamin D [28,38,39]. Autophagy is an essential process for cell homeostasis [40], and plays a key role in cellular responses to oxidative stress and inflammation [41,42,43,44].

## 2. Anti-inflammatory Properties of Vitamin D

Serum vitamin D levels are inversely associated with interleukin (IL)-6 and high-sensitivity C-reactive protein (hsCRP) levels in stroke individuals, consistent with a potential anti-inflammatory role of vitamin D after stroke [45,46]. Experimental evidence has shown that activation of VDR by calcitriol has immunomodulatory actions [47,48,49,50] and prevents leukocyte recruitment to injured tissues [49,51,52].

Calcitriol exerts its immunomodulatory actions through a variety of mechanisms. It down-regulates nuclear factor kappa-light-chain-enhancer of activated B cells (NF-κB), a transcription factor involved in inflammatory gene expression in lymphocytes [53], and can inhibit its activation by reducing DNA binding of NF-κB [54]. Vitamin D can also dampen inflammation after myocardial injury by inhibiting the RhoA/Rho-associated protein kinase (ROCK)/NF-κB pathway [55]. Furthermore, the VDR/RXR complex binds to the nuclear factor of activated T cells (NFAT) binding site of the IL-2 promoter and inhibits NFAT activity in T cells, thus blocking T cell proliferation [56] and leading to reduced expression of IL-17A [48]. Calcitriol can modulate the phenotype of T cells by downregulating Janus kinase (JAK)–signal transducer and activator of transcription (STAT) signaling, which is critical for the development of pathogenic T helper (Th) cells, such as Th1 and Th17 cells [57,58,59], gamma-delta (γδ) T cells [60], and their cytokine production [61]. Moreover, calcitriol can promote the polarization of anti-inflammatory Th2 [62] and T regulatory (Treg) cells [63], thus inhibiting inflammation-driven injury.

**Figure 2:**
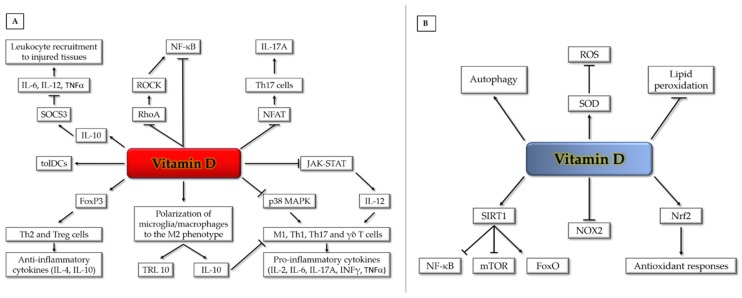
Vitamin D pleiotropic effects on anti-inflammatory (**A**) and antioxidant (**B**) signaling pathways and mechanisms (see text for details). FoxO (Forkhead box-O); FoxP (Forkhead box-P);; JAK (Janus kinase); IL (interleukin); INFγ (interferon-gamma); MAPK (mitogen-activated protein kinase); mTOR (mammalian target of rapamycin); NFAT (nuclear factor of activated T cells); NF-κB (nuclear factor kappa-light-chain-enhancer of activated B cells); NOX (NADPH oxidase); Nrf2 (nuclear factor erythroid 2-related factor 2); RhoA (RhoA GTPase); ROCK (Rho-associated protein kinase); ROS (reactive oxygen species); SIRT1 (Sirtuin 1); SOCS3 (suppressor of cytokine signaling-3); SOD (superoxide dismutase); STAT (signal transducer and activator of transcription); TLR (toll-like receptor); TNFα (tumor necrosis factor alpha); γδ T (gamma-delta T cells); M1 (M1 macrophages); Th (T helper cells); tolDCs (tolerogenic dendritic cells); Treg (regulatory T cells).

Lastly, calcitriol can promote the generation of tolerogenic dendritic cells [64,65] and prevent the release of pro-inflammatory cytokines from monocytes/macrophages via inhibition of the p38 MAP kinase [49,66]. Likewise, it can inhibit atherosclerosis by promoting polarization of macrophages to an M2-phenotype [67]. Calcitriol can similarly exert an anti-inflammatory action on human microglia by facilitating M2 differentiation and upregulation of the anti-inflammatory toll-like receptor (TLR) 10 [68]. Furthermore, calcitriol reduces the expression of pro-inflammatory cytokines via promoting induction of the suppressor of cytokine signaling-3 (SOCS3) by IL-10 [69] (Figure 2A).

Inflammation is well established to be a key contributing factor in secondary brain injury after ischemic stroke, making this process a rational target for new therapies [70]. We recently explored the effect of experimentally elevating levels of vitamin D in vitamin D-replete mice [50], analogous to high-dose supplementation regimes in humans [71,72]. Acute supplementation of vitamin D reduced infarct volume by ~50%, reduced the gene expression of pro-inflammatory mediators, and increased the expression of the T regulatory cell marker, Forkhead box-P3 (FoxP3) [50]. Our findings not only demonstrated a direct impact of vitamin D on the degree of inflammation and secondary brain injury that developed following stroke, but indicated that acute calcitriol supplementation can actually limit the resulting injury, even in vitamin D-sufficient individuals [50].

## 3. Antioxidant Properties of Vitamin D

Among its potential pleiotropic roles in human health, it has been suggested that the active form of vitamin D may act as a membrane antioxidant, protecting cell membranes against free radical-induced lipid peroxidation through interaction with phospholipid fatty acid side chains, in order to increase stabilization of the membrane structure [73]. Indeed, there is evidence that vitamin D can be as effective as vitamin E, a major dietary lipid-soluble antioxidant, in reducing lipid peroxidation and inducing the activity of reactive oxygen species (ROS) scavenging enzymes, such as superoxide dismutase (SOD) [74]. Moreover, it has been reported that vitamin D can stimulate sirtuin 1 (SIRT1), a protein deacetylase known to exert cardioprotective effects, by increasing autophagy and mitochondrial function via inhibition of the mTOR pathway and reducing oxidative stress and inflammatory responses, via activation of FoxO-dependent antioxidant pathways and inhibition of NF-κB signaling, respectively [75,76,77,78]. Furthermore, vitamin D has been shown to exert an antioxidant role by VDR-mediated transcriptional downregulation of NOX2, a major isoform of NADPH oxidase [79], and upregulation of Nrf2, a master inducer of antioxidant responses [80,81,82] (Figure 2B). Such an action is consistent with the upregulation of critical biomarkers of oxidative stress, including 8-hydroxy-2’-deoxyguanosine (8-OHdG) observed in VDR knockout mice [83].

More generally, it appears that vitamin D acts as a guardian of cellular homeostasis and protects from oxidative stress in various cell types, including human endothelial cells, through its ability to regulate crosstalk between redox signaling and autophagy [27,28,38,84]. Accordingly, whereas it is now well-established that autophagy serves as an essential cellular antioxidant system by removing damaged or dysfunctional proteins and organelles [40,41,85], endothelial cell viability can be enhanced by pre-treatment with vitamin D before the induction of oxidative stress [27]. Furthermore, despite some controversy over the clinical relevance of the antioxidant properties of vitamin D [86], recent studies have shown that vitamin D supplementation increases basal levels of autophagy and decreases oxidative stress parameters, suggesting a therapeutic potential in oxidative stress-related diseases [39,85,87,88,89,90,91,92]. Indeed, vitamin D is known to exert antioxidant properties in the endothelium [27,93,94], and vitamin D supplementation is reported to decrease the burden of pathological vascular phenotypes related to oxidative stress in a mouse model of a cerebrovascular disease [95].

Nevertheless, an individual’s response to a given dose of vitamin D may be influenced by multiple factors, including genetic modifiers, as analysis of primary tissues from vitamin D intervention studies have indicated large interindividual variation for the efficacy of vitamin D supplementation, an issue that should be carefully considered in clinical management [12,96,97,98].

## 4. Determinants of Vitamin D Status and Related Health Outcomes

Serum concentration of 25(OH)D, the main circulating metabolite of vitamin D, is the best indicator of vitamin D status, as it has a quite long half-life of 15 days and reflects both cutaneous synthesis and dietary intake from foods and supplements. In contrast, circulating 1,25(OH)_2_D is generally not considered a good biomarker of vitamin D status, mainly because it has a short half-life of 15 h, and its levels do not reflect strictly cutaneous synthesis and dietary intake. Although there is currently no worldwide consensus on the optimal vitamin D status, serum 25(OH)D levels ≥ 75 nmol/L (≥ 30 ng/mL) are generally considered adequate for overall health, whereas levels between 50 and 75 nmol/L (20–30 ng/mL) indicate “insufficiency”, and levels < 50 nmol/L (< 20 ng/mL) indicate “deficiency”. On the other hand, there is emerging evidence that serum 25(OH)D concentrations > 150 nmol/L (> 60 ng/mL) are associated with potential adverse effects [99,100].

Multiple factors, including environmental and genetic determinants and their interactions, account for variation in serum 25(OH)D levels and health consequences. The main source of vitamin D is cutaneous biosynthesis induced by skin exposure to UVB light from the sun. Thus, baseline levels of serum 25(OH)D can vary significantly according to both seasonal and geographical variations, and are typically lower at the end of winter, especially when living at high latitudes (greater than 37th parallel north or south of the equator). Indeed, people who live in the far northern and southern hemispheres often cannot make any vitamin D_3_ in their skin for up to 6 months of the year, and are therefore at relatively greater risk for vitamin D deficiency [101,102]. However, intake of recommended dietary allowance (RDA) levels of vitamin D from foods or supplements, as well as established food fortification programs, such as those in Finland, Sweden, the United States, Canada, and Australia, can completely compensate for the lack of cutaneous vitamin D biosynthesis due to insufficient sunlight exposure [102,103,104,105,106]. More generally, several recommendation-setting bodies have provided guidelines for the maintenance of optimal vitamin D status and the treatment of vitamin deficiency, suggesting that ≥ 75 nmol/L (≥ 30 ng/mL) is the optimal baseline 25(OH)D concentration to be maintained during the year through adequate dosage regimens of vitamin D intake from food or supplements, in order to prevent a drop below 50 nmol/L due to seasonal and geographical influences [99].

Besides seasonal, geographic, and lifestyle variations, a further factor that impinges on circulating 25(OH)D levels is the skin pigmentation-related efficiency of sunlight-induced vitamin D production. Indeed, high levels of the protective skin-darkening pigment melanin reduce the skin’s ability to produce vitamin D from sunlight. Consistently, there is evidence that skin color change between summer and winter predicts seasonal 25(OH)D change [104,107], as well as that much of the variation in serum 25(OH)D concentration between racial/ethnic groups may be attributed to skin color [108]. Overall, sun exposure, vitamin D intake, demographics, and lifestyle, as well as their potential interactions, are important determinants of vitamin D status, and could therefore have a significant impact both on its role in pathophysiological mechanisms and its therapeutic applications.

Regarding genetic determinants, numerous genome-wide association studies (GWAS) have identified multiple genetic modifiers of vitamin D serum levels and biological effects, including single-nucleotide polymorphisms (SNPs) in genes coding for key proteins involved in vitamin D metabolism, transport, and signaling, which could be considered in the clinical management with vitamin D. In particular, various polymorphisms in genes encoding 7-dehydrocholesterol reductase (DHCR7, which shunts vitamin D precursors toward cholesterol biosynthesis); cytochrome P450 vitamin D hydroxylases, including CYP2R1 (25-hydroxylase), CYP27B1 (1α-hydroxylase), and CYP24A1 (24-hydroxylase); vitamin D binding protein (VDBP, also known as GC globulin); and vitamin D receptor (VDR) were found to be statistically significantly associated with inter-individual variations in serum 25(OH)D and 1,25(OH)2D levels, either at baseline or after vitamin D supplementation [109,110], as well as with susceptibility to various human diseases [98,110,111,112,113,114]. More specifically, distinct randomized controlled trials (RCTs) and systematic reviews/meta-analyses have shown that genetic variants in CYP2R1 (rs10766197, rs10741657, rs12794714, rs1562902, rs2060793), CYP24A1 (rs2209314, rs2762939, rs6013897), and VDBP (rs7041, rs4588) are either associated with baseline serum 25(OH)D levels or modify the efficacy of vitamin D supplementation in increasing such levels [109,110,114,115,116]. On the other hand, polymorphisms in CYP27B1 (rs703842, rs10877012, rs4646536) and VDR (rs2228570, rs7975232, rs1544410) were most commonly reported to be associated with health outcomes, including susceptibility to metabolic, inflammatory, autoimmune, and infectious diseases [98,110,111,112,116].

While further validation studies with large sample sizes and controlled confounding factors are still needed, these findings point to a potential interplay between vitamin D deficiency and polymorphisms in vitamin D-related genes, suggesting important implications for achieving optimal vitamin D status and health outcomes in individuals with different genetic backgrounds.

Overall, genetic and nongenetic determinants of vitamin D status are significant predictors of its health outcomes, and may have an important impact on susceptibility to various human diseases. Indeed, there is already clear evidence that both seasonal variations and genetic modifiers of vitamin D metabolism and functions are independently associated with inter-individual variability with regard to the risk of developing cardiovascular disorders, including severe cerebrovascular diseases [117,118], as described in more detail below.

## 5. Vitamin D Deficiency and Its Impact on Cerebrovascular Diseases

It is noteworthy that a meta-analysis of pooled data from 32 studies recently found that serum 25(OH)D concentrations < 30 ng/mL were associated with higher all-cause mortality than concentrations > 30 ng/mL [119]. Specifically, despite some conflicting results, there is evidence that low solar UVB exposure and low serum 25(OH)D levels are associated with an increased risk of CVD, as well as that CVD mortality is about double in older individuals with deficient 25(OH)D concentrations compared with age-matched individuals with adequate 25(OH)D concentrations (> 30 ng/mL) [120,121,122,123,124]. Indeed, accumulating evidence suggests that vitamin D insufficiency or deficiency may adversely affect the cardiovascular system through multiple effects. Levels of the pro-hormone cholecalciferol are maintained either through dietary consumption or epidermal synthesis following exposure to ultraviolet light [125]. Nonetheless, “vitamin D insufficiency”, defined as 20–29 ng/mL of serum 25(OH)D, is endemic in humans, with more than a billion people affected worldwide, and may require public health actions, such as systematic vitamin D food fortification [106,126]. Moreover, “vitamin D deficiency” (< 20 ng/mL) is prevalent in almost half of the healthy population of developed countries [126,127], is common in patients with CVD [127], and is independently associated with a higher risk for future cardiovascular events [126,128]. Furthermore, vitamin D deficiency is likely to be associated with advanced age, darker skin pigmentation, less sunlight exposure, and low dietary intake of vitamin D [129], and has been linked to an increased risk of age-related morbidities that include neurodegenerative diseases and cerebrovascular dysfunctions [130,131,132].

Epidemiological evidence suggests an association of vitamin D insufficiency with endothelial dysfunction in healthy and pathological conditions [133,134]. Indeed, calcitriol acts as a direct transcriptional regulator of endothelial nitric oxide (NO) synthase (eNOS), and can promote normalization of eNOS mRNA expression and enzymatic activity in experimental atherosclerosis [135]. Mice carrying mutated, functionally inactive VDR exhibit increased arterial stiffness, endothelial dysfunction, and lower NO bioavailability due to reduced eNOS expression [136]. Mice with endothelial-specific deletion of the VDR exhibit reduced eNOS expression, impaired endothelium-dependent vasorelaxation, and an augmented pressor effect of angiotensin II [137]. Furthermore, vitamin D can exert powerful immunomodulatory actions to modify the immune response to injury during various diseases, including atherosclerosis [64,138], cancer [25], asthma [139], and stroke [50]. In particular, vitamin D can attenuate inflammatory responses and promote protein homeostasis via modulating the NF-κB and unfolded protein response (UPR) pathways, respectively [140,141]. In addition, vitamin D can regulate matrix homeostasis through the modulation of distinct matrix metalloproteinases (MMPs) and tissue inhibitors of metalloproteinases (TIMPs) [142], consistent with its deficiency being critical in major cerebrovascular diseases, including brain aneurysms, cerebrovascular malformations, and stroke, where matrix destabilization is significant [118,143,144]. Overall, it is plausible that the impaired endothelial function that may accompany low circulating vitamin D levels contributes to an increased risk of cerebrovascular diseases and mortality. Indeed, there is increasing interest in how vitamin D levels might influence the onset and severity of cerebrovascular diseases, including stroke [145,146,147,148,149,150] and cerebrovascular malformations [43,95,117,128,151,152].

### 5.1. Vitamin D Deficiency and Stroke

Stroke is a crippling cardiovascular event that accounts for 5–10% of all deaths, and is the leading cause of serious long-term disability, with > 50% of survivors discharged into care [153]. About 85% of strokes are caused by a clot formed in a cerebral artery in which a dysfunctional endothelium would be typically present [153,154]. Vitamin D deficiency is particularly frequent in people who have suffered a stroke, which is commonly associated with advanced age, limited mobility, decreased sunlight exposure, and higher prevalence of malnutrition [155]. Meta-analyses have found that a low vitamin D level contributes to a ~50% increased risk of incident stroke [156], and that there is a stepwise increase in stroke risk with decreasing plasma vitamin D level [145].

Clinical vitamin D status appears to influence the incidence, impact, and recovery from ischemic stroke. Vitamin D deficiency was recently found to be a risk factor for incident stroke, and the strength of this association does not appear to differ by race [157,158]. Strikingly, in a prospective study of 58,646 healthy adults with median follow-up period of 19.3 years, vitamin D intake was inversely associated with the risk of mortality from stroke [159]. Furthermore, low serum levels of vitamin D at admission have been proposed as an independent prognostic biomarker for greater stroke severity [160,161], a larger infarct volume in the acute phase [162], a poorer functional outcome at discharge [160,161], a higher incidence of cognitive impairment at one month [150], a higher risk of death at one or two years [160,163], and a greater risk of early recurrent stroke [149,163]. There is also evidence that high circulating levels of vitamin D are associated with less cognitive impairment among stroke patients, manifested as greater functional improvement during rehabilitation [147,164]. The relationship between plasma vitamin D levels and functional outcome from stroke also applies to patients who have received intravenous thrombolysis [148], consistent with the possibility that improving vitamin D status after stroke might provide benefits in addition to those from reperfusion. In a prospective population-based study of > 9300 participants, an association was found between vitamin D and prevalent stroke, but only severe vitamin D deficiency was associated with incident stroke, with the authors concluding that lower vitamin D levels may not lead to a higher stroke risk, but may instead be a consequence of stroke [165].

Furthermore, there is evidence that a sufficient level of vitamin D could exert several neuroprotective actions, including reduction of oxidative stress [166], regulation of neuronal inflammation and death from stroke [50], and fewer neurogenerative disorders [167]. These findings raise the possibility that low vitamin D serum levels, a treatable risk factor, might be targeted for the reduction of disability among stroke sufferers. Indeed, a study to assess the long-term effect of supplementation of vitamin D in ischemic stroke patients with low vitamin D levels found a significant improvement in the outcome after three months [168]. Nevertheless, the value of vitamin D supplementation in preventing stroke is still unclear, as a recent meta-analysis of randomized clinical trials conducted in more than 80,000 patients found no clinical benefit of vitamin D supplementation in reducing the incidence of (as opposed to outcome from) major cardiovascular events, including stroke and cardiovascular death [169]. Therefore, it is possible that vitamin D supplementation is less effective for preventing cardiovascular events than for limiting post-stroke injury and improving outcome.

### 5.2. Vitamin D Deficiency and CCM Disease

Cerebral cavernous malformation (CCM), also referred to as cavernous angioma or cavernoma, is a significant vascular disease of genetic origin. CCM lesions mostly occur within the central nervous system, and involve closely clustered, abnormally dilated and leaky capillaries, which are characterized by a thin endothelium devoid of normal vessel wall components [43,170]. These lesions have a prevalence of 0.5% in the general population, and can be detected by magnetic resonance imaging (MRI) as single or multiple mulberry-like vascular sinusoids of varying size and locations. Most CCM lesions are clinically and biologically inactive; however, in 30% of cases, they result in various clinical symptoms, including focal neurological deficits, recurrent headaches, stroke, intracerebral hemorrhage (ICH), and seizures. Clinical presentation can occur at any age and at varying levels of severity.

Genetic studies in CCM patient cohorts have led to the identification of three disease genes, *CCM1*/*KRIT1*, *CCM2*, and *CCM3*, which have been implicated in all major mechanisms of vascular integrity maintenance and endothelial barrier function, including the coordination of redox signaling and autophagy governing cell homeostasis and stress responses [43,171,172,173,174,175,176,177,178,179,180]. Consistent with such pleiotropic functions, accumulated evidence from animal models and patient cohorts has demonstrated that loss-of-function mutations of CCM genes only predispose to the development of CCM disease. CCM may eventually occur with incomplete penetrance and highly variable expressivity, depending on additional local factors, such as oxidative stress, inflammation, and sensitivity to stress [43,118,180,181].

Vitamin D deficiency is one of the risk factors that may impact the susceptibility to local oxidative stress and inflammation, and thereby health outcomes for vulnerable carriers of mutations in CCM genes. Indeed, whereas meta-analyses of general observational studies have consistently associated vitamin D deficiency with an increased risk for inflammatory and CVDs [120,182,183,184,185,186], specific epidemiological studies have shown that vitamin D serum levels, including throughout their seasonal variation, are negatively associated with risk for CCM disease onset and severity [117,151,152]. Moreover, a genome-wide association study (GWAS) in a large cohort of CCM patients sharing a common founder mutation in the KRIT1 gene (Q455X) identified a correlation between CCM disease severity and SNPs in genes involved in vitamin D metabolism and function, including CYP and MMP family members. This suggested that SNPs in these genes can act as genetic susceptibility factors and modifiers of CCM disease penetrance and expressivity [118]. In particular, CYP27A1 and CYP27B1, two essential players in the vitamin D signaling system [187,188], were among the putative genetic modifiers of the CYP family that appear to influence interindividual differences in the susceptibility to develop the most severe disease phenotypes [118]. Furthermore, whereas various vascular protective actions of vitamin D have been shown [27,120,128], an unbiased screening strategy for identifying repurposed drugs for CCM disease treatment has led to the discovery of vitamin D as a major preventive and therapeutic candidate, along with tempol (a scavenger of superoxide) [95]. In particular, as both compounds were effective in decreasing cerebrovascular lesion burden in a mouse model of CCM disease by ≈ 50%, it was suggested that their effectiveness was related to their shared ability to promote endothelial stability through specific antioxidant, anti-inflammatory, and pro-autophagic activities [43,95,128,173,174,175]. Indeed, as mentioned above, vitamin D can enhance endothelial barrier function and inhibit peripheral vascular diseases by stimulating autophagy and limiting oxidative stress and inflammatory events, including the production of ROS, lipopolysaccharides, and inflammatory cytokines [27,35,93,94,128,189,190].

Thus, the emerging evidence indicates that defective autophagy and redox imbalance play a major role in the genesis and progression of CCM lesions [43,175,180], as well as that vitamin D exerts significant protective effects by counteracting such pathogenetic mechanisms [43,95,174,175]. Taken together, these findings suggest that vitamin D should be considered within the recently proposed drug combination therapy approaches aimed at gaining additive or synergistic effects for a more effective treatment of CCM disease and associated comorbidities [180,191,192].

## 6. Conclusions and Perspectives

There is now considerable evidence that, besides regulating calcium homeostasis, vitamin D influences other fundamental biological processes, such as autophagy, mitochondrial function, redox homeostasis and signaling, epigenetic changes, and oxidative stress and inflammatory responses. Serum vitamin D levels, as well as polymorphisms in VDR and CYP enzymes involved in the three main steps of vitamin D metabolism (25-hydroxylation, 1α-hydroxylation, and 24-hydroxylation) [2], have been increasingly associated with the incidence of various human diseases, including cancer and inflammatory, autoimmune, and neurodegenerative diseases, as well as CVDs [35,98,116,193] This is consistent with findings in VDR- and 1α-hydroxylase-deficient mice [194].

**Figure 3:**
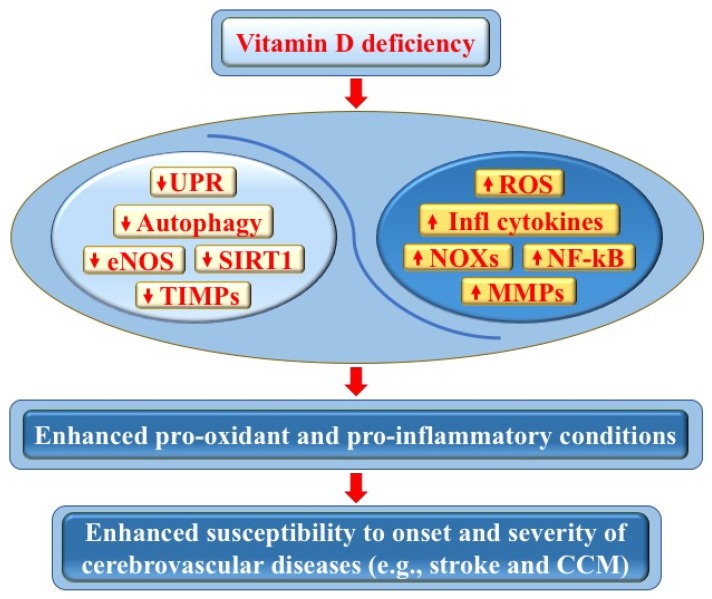
Vitamin D deficiency and its impact on cerebrovascular diseases. Vitamin D deficiency may adversely affect endothelial cell function and vascular homeostasis through pleiotropic pro-oxidant and pro-inflammatory effects, including the downregulation of autophagy, unfolded protein response (UPR), endothelial nitric oxide synthase (eNOS), sirtuin 1 (SIRT1), and tissue inhibitors of metalloproteinases (TIMPs), as well as the upregulation of NADPH oxidases (NOXs), reactive oxygen species (ROS), nuclear factor kappa-light-chain-enhancer of activated B cells (NF-κB), inflammatory cytokines, and matrix metalloproteinases (MMPs). In turn, these effects can promote pro-oxidant and pro-inflammatory conditions, as well as an enhanced tissue sensitivity to oxidative stress and inflammatory events, with consequent increased susceptibility to the onset and severity of cerebrovascular diseases, including stroke and cerebral cavernous malformation (CCM) disease (see text for details).

This review has focused on two of the major cerebrovascular diseases that are demonstrably connected to vitamin D deficiency: stroke and CCM disease. We have highlighted underlying pleiotropic pro-oxidant and pro-inflammatory molecular mechanisms, as well as the likely importance of maintaining an optimal vitamin D homeostasis for mitigating the inflammation–oxidative stress cycle that may exacerbate these cerebrovascular diseases (Figure 3). Indeed, while vitamin D deficiency is endemic in the population, it is easy to screen for and can be readily and inexpensively treated by dietary supplementation and modest sunlight exposure. In particular, whereas a low serum level of vitamin D is associated with higher risk of stroke and negatively impacts recovery and mortality from stroke, preclinical data suggest that acute administration of vitamin D can limit infarct progression by modulating post-stroke brain inflammation. Moreover, whereas it is now established that vitamin D modulates endothelial homeostasis and barrier function [128], a potential benefit of vitamin D in preventing or limiting the onset and severity of CCM disease has clearly emerged from both epidemiological studies and animal models [43,95,117,151,152].

On the other hand, the therapeutic effect of vitamin D supplementation remains controversial, as there is still some inconsistency in the conclusions of distinct RCTs and meta-analyses concerning its beneficial effects on oxidative stress biomarkers and CVD outcomes, including stroke, or on mortality [25,119,159,161,168,169,195,196,197,198,199]. Such controversy is akin to that regarding the putative beneficial effects of antioxidants in optimizing health [200], with the reported discrepancies likely due to the complexity of the systems and existence of multiple confounding factors. Indeed, a careful analysis of existing RTC and related meta-analysis studies on the effects of vitamin D supplementation reveals several methodological limitations, including the use of diverse study populations, different doses of vitamin D with or without Ca^2+^, different durations of supplementation and follow-up, different baseline and acquired circulating 25(OH)D concentrations, and different study outcome parameters [99,201]. Furthermore, significant discrepancies could result from the large inter-individual variation in genetic determinants of the efficacy of vitamin D supplementation [12,109,110], as well as its context-dependent effects on mechanisms in endothelial cells, including autophagy [202]. Consistent with these potential shortcomings in evaluating the effects of vitamin D supplementation, there remains much controversy and an open debate about the reliability of either RCTs or their meta-analyses [99,203,204].

In contrast, the outcomes of a large number of experimental studies have consistently shown that vitamin D influences most of the risk factors and molecular mechanisms associated with CVDs, suggesting that its use in the clinical setting should be considered for the prevention of the onset, progression, and severity of these diseases.

In conclusion, while the benefit of vitamin D supplementation on cerebrovascular outcome requires deeper study, it may represent a novel approach for limiting the overall impact of acute stroke and CCM disease. Therefore, we suggest that it is now essential both to implement accurate screenings and large, well-powered RCTs for investigating vitamin D deficiency and supplementation in stroke and CCM patients, in order to more fully characterize the molecular mechanisms underlying the effects of vitamin D. In particular, a better understanding of the emerging major role of autophagy in the multiple health-promoting effects of vitamin D [28] is needed, as well as the role of factors that influence the activity of CYP vitamin D-metabolizing hydroxylases and local production of vitamin D’s hormonally active form, calcitriol [1,2]. Such knowledge may lead to the design of drug combinations and multitargeting therapeutic strategies for the more effective treatment of diseases associated with vitamin D deficiency.
